# Cauda equina syndrome as the initial presenting clinical feature of medulloblastoma: a case report

**DOI:** 10.1186/1752-1947-6-135

**Published:** 2012-05-23

**Authors:** Faisal Al-Otaibi, Anwar Ul-Haq, Hindi Al-Hindi, Amani Al Kofide, Essam Al Shail

**Affiliations:** 1 Division of Neurosurgery, Neurosciences Department, King Faisal Specialist Hospital and Research Center, PO Box 3354, Riyadh, 11211, Saudi Arabia; 2 Department of Pathology and Laboratory Medicine, King Faisal Specialist Hospital and Research Center, Riyadh, Saudi Arabia; 3 Division of Pediatric Hematology/Oncology, Oncology Center, King Faisal Specialist Hospital and Research Center, Riyadh, Saudi Arabia

**Keywords:** Cauda equina syndrome, Drop metastasis, Medulloblastoma, Paraparesis

## Abstract

**Introduction:**

Medulloblastoma is one of the most common pediatric brain malignancies. The usual presenting clinical features are related to posterior fossa syndrome or/and hydrocephalus. Cauda equina syndrome is a very rare presentation for this disease.

**Case presentation:**

We describe the case of a three-year-old boy with cauda equina syndrome as the initial presenting clinical feature for medulloblastoma. He was initially diagnosed as having a spinal tumor by magnetic resonance imaging scan. Subsequently, a cranial magnetic resonance imaging scan revealed a posterior fossa tumor with features of dissemination. He had substantial improvement after treatment. This case report is complemented by a literature review related to this unusual presentation.

**Conclusions:**

Medulloblastoma primarily presenting with cauda equina syndrome is very rare. However, spinal drop metastasis should be considered in the pediatric age group to avoid suboptimal management.

## Background

Medulloblastoma is the second most common posterior fossa tumor in children. It accounts for 40% of posterior fossa tumors in the pediatric age group [1]. The clinical presentation of this malignant tumor is usually related to the posterior fossa tumor mass effect and/or obstructive hydrocephalus. Cerebrospinal fluid (CSF) dissemination to the cranio-spinal axis occurs in 30% to 40% of cases [2]. However, medulloblastoma primarily presenting with symptoms related to spinal metastasis is extremely rare [2,3]. To date, there are only a limited number of cases that have been reported in the literature. Here, we report the case of a child with medulloblastoma who presented with progressive paraparesis caused by cauda equina drop metastasis without symptoms related to the primary intracranial tumor.

## Case presentation

Our patient was a three-year-old Arabic boy who presented to a peripheral medical center with a two-month history of lower back pain followed by progressive paraparesis. He had no symptomatology related to posterior fossa syndrome or increased intracranial pressure. Six weeks after the start of his symptoms, he developed urinary and bowel incontinence. During the physical examination, the boy was fully awake and cooperative. All cranial nerves were intact. His pupils were 2 mm, bilaterally equal and normally reacting to light. He had full extra-ocular movement with no nystagmus. There was no papilloedema. An examination of the upper extremities was normal. He had symmetrical lower limb weakness with muscle group power grade 1/5 distally and 3/5 proximally associated with hypotonia, absent reflexes and diminished sensations up to the L1 dermatome. His anal tone was reduced. Cauda equina syndrome was suspected, for which he underwent a magnetic resonance imaging (MRI) scan of the spine that revealed spinal canal filling and lesions in the thoracic and lumbar regions. At that stage, he was referred to our institution as a patient with a spinal tumor.

**Figure 1:**
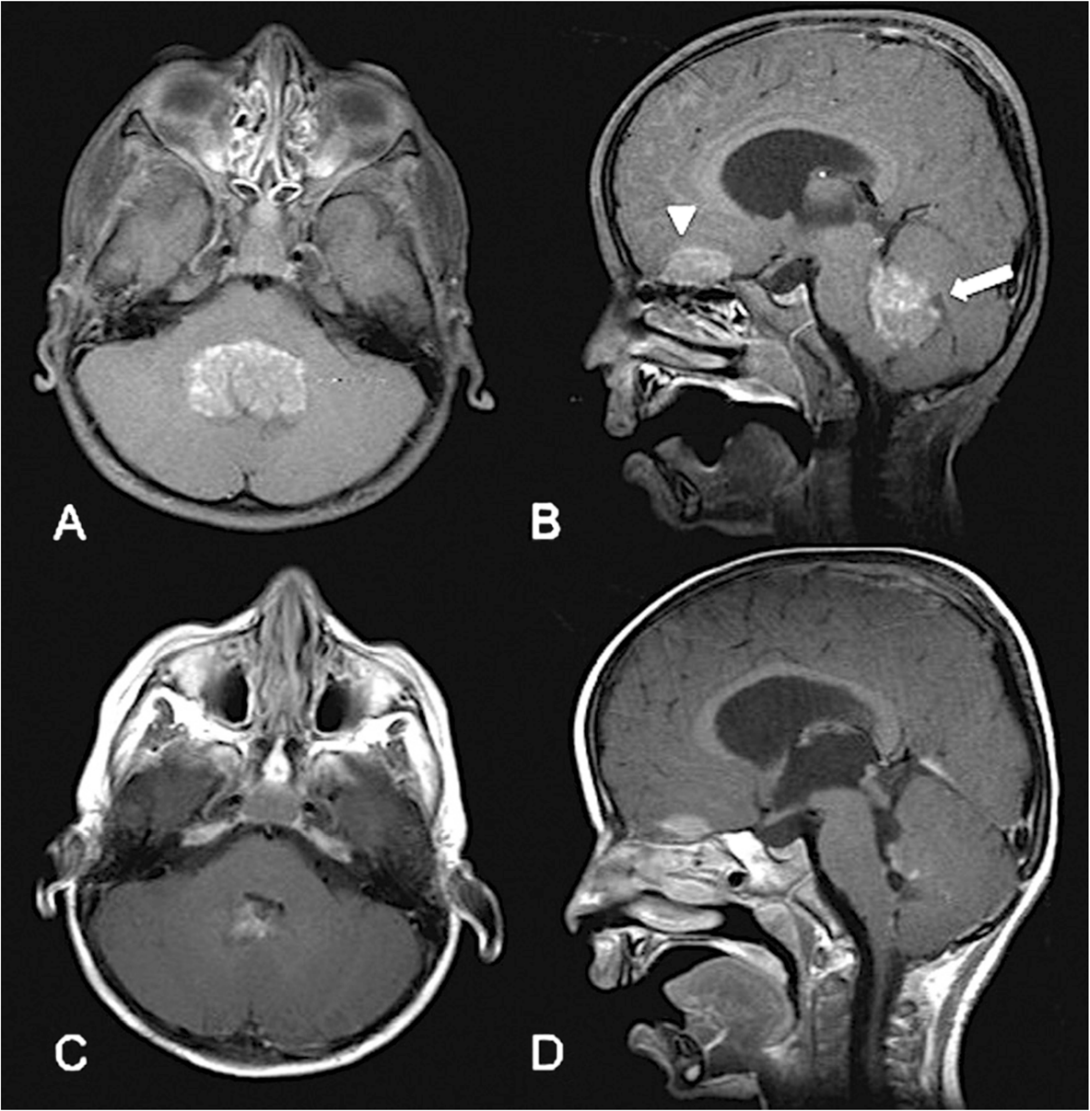
**(A) Axial magnetic resonance imaging T1-weighted image with gadolinium, showing a posterior fossa tumor.** (**B**) Sagittal magnetic resonance imaging T1-weighted image with gadolinium showing posterior fossa tumor (arrow) and frontobasal tumor (arrowhead), suggestive of cerebrospinal fluid (CSF) metastasis. (**C**,**D**) Post-treatment, one-year follow-up axial and sagittal magnetic resonance imaging revealed regression of the disease.

**Figure 2:**
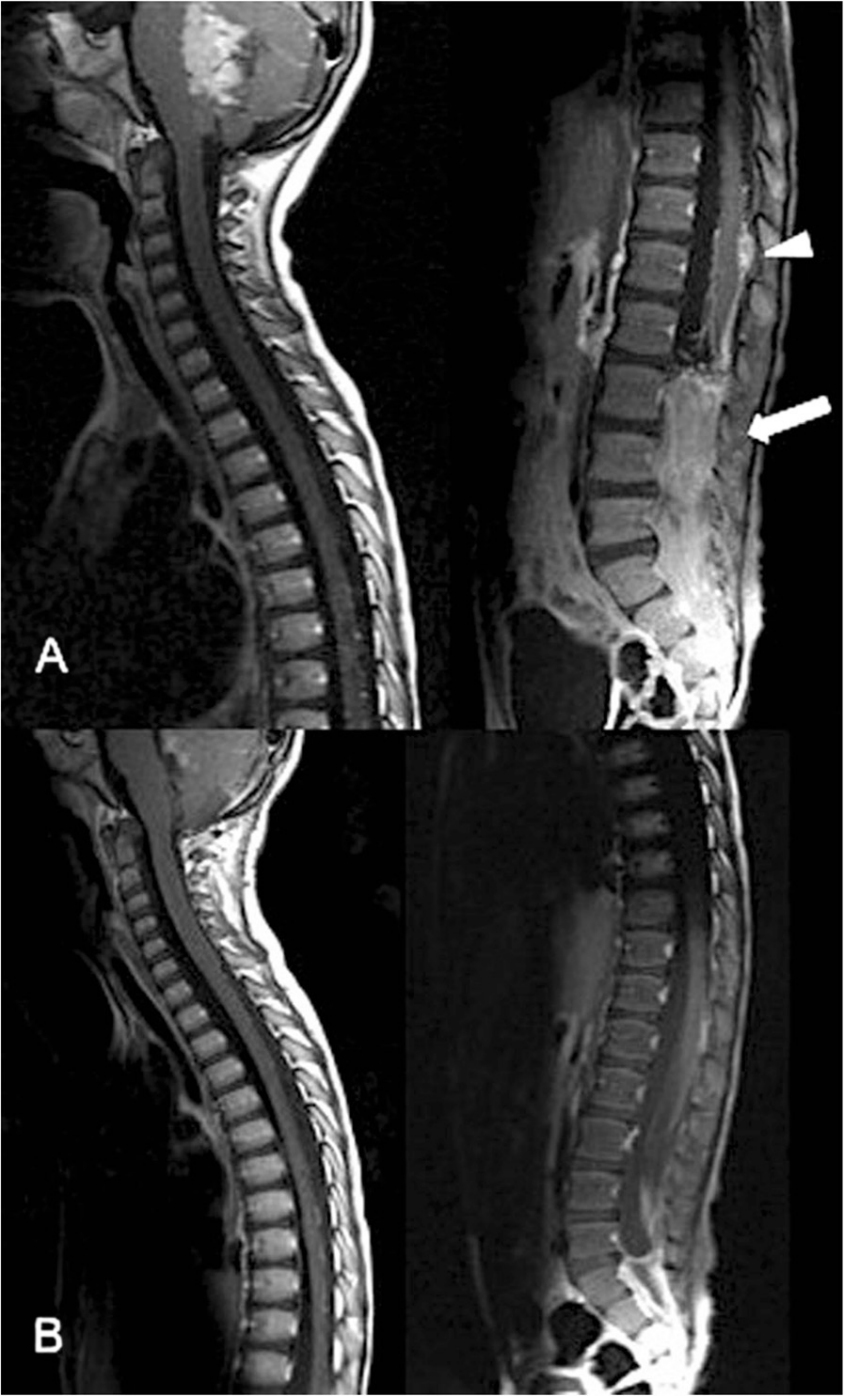
**(A) Pre-treatment spinal T1-weighted image with gadolinium demonstrating a nodular extra-medullary enhancement (arrowhead) and spinal block in the lumbar region (arrow).** (**B**) Post-treatment one-year follow-up spinal magnetic resonance imaging revealed regression of the disease.

**Figure 3:**
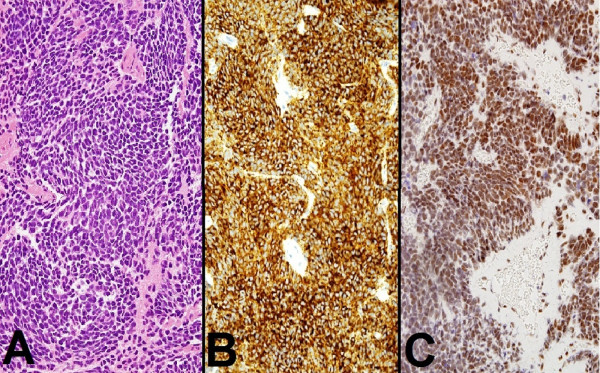
Morphology of classic medulloblastoma as shown in (A) (hematoxylin and eosin stain; original magnification, 200×) with strong and uniform expression of synaptophysin (B) (Novocastra™, clone 27 G12, 1:100; original magnification, 200×) and BAF47 (C) (BD Biosciences, clone 25, 1:200; original magnification, 200×).

Upon reviewing his spinal MRI, drop metastasis was suspected. A cranio-spinal MRI scan was performed, which showed a posterior fossa tumor associated with radiographic features of intracranial and spinal metastasis, (Figures 1A, B and 2A). This MRI feature was suggestive of medulloblastoma. Given the fact that the thoracolumbar pathology is mainly a diffuse process affecting cauda equina roots, which is probably not amenable to surgical removal, we decided to direct the surgical treatment to the primary posterior fossa tumor only. Therefore, he underwent a suboccipital craniotomy and subtotal resection of the posterior fossa tumor. Neuropathological examination of the resected specimen revealed features of classic medulloblastoma. The cells had primitive nuclei, which were packed without signs of differentiation (Figure 3A). Also, the tumor lacked any anaplastic or large cell features. The tumor cells were uniformly expressing synaptophysin (Novocastra™, clone 27 G12, 1:100, Newcastle, UK) (Figure 3B). Since our patient was young and the clinical course was atypical, immunostaining for integrase interactor 1 (INI-1) protein (BAF47; BD Biosciences, clone 25, 1:200) was also performed and the neoplastic cells were uniformly immunoreactive to it (Figure 3C). This practically excludes atypical teratoid/rhabdoid tumor as a diagnostic possibility. Subsequently, our patient received cranio-spinal axis radiotherapy (36 Gy) and 18 Gy boost to the posterior fossa. Adjunctive chemotherapy was given, which consisted of vincristine, cisplatin, and CCNU (lomustine).

During his treatment course he received rehabilitation therapy. Three months after his initial treatment, he had substantial improvement in his overall neurological functions. He started to walk without assistance and was able to control his bladder and bowel functions. A neuroaxis follow-up MRI after one year showed regression of his cranial and spinal disease (Figures 1C, D and 2B). At the time of writing this report, he has retained good neurological function.

## Discussion

The usual presenting symptoms of medulloblastoma are headache, vomiting and ataxia [2,4]. Although CSF dissemination to the cranio-spinal axis can happen in 30% to 40% of cases, initial presentation with spinal cord compression is quite rare [1,2,5]. Pezeshkpour and colleagues analyzed more than 18,000 primary central nervous system tumors and found that only 0.01% of them had drop spinal metastasis, which had caused the presenting symptoms [6]. At the time of diagnosis around 10% to 35% of the cases had extra-medullary intradural metastasis, however, their main presenting symptoms were due to the primary intracranial tumor [3]. In 1986, Wang *et al.,* reported the case of a 28-year-old woman with thoracic extra-medullary medulloblastoma metastasis causing her to have progressive weakness as the initial presenting symptoms (Table 1) [7]. More recently, Lee and colleagues from China described a case with acute paraplegia caused by drop spinal metastasis [8]. Stanley and colleagues, in 1988, reported on 34 patients with medulloblastoma [5]. Fifteen of those had a positive result on myelogram for spinal metastasis and only one patient suffered from lower limb weakness related to spinal pathology; however, it was not the initial presenting symptom. Our group, in 1996, reported on the outcomes of 149 patients who received treatment at our institution. None of those had presented initially with symptoms related to spinal metastasis at time of diagnosis [9]. Two adult patients have been reported in non-English language articles: one with cauda equine syndrome and the other with radiculitis features as the presenting clinical features of medulloblastoma [10,11].

**Table 1:** Selected reported cases of cauda equine syndrome as the main initial presenting clinical features

**Author (year) and reference**	**Age**	**Gender**	**Clinical presentation**
Wang [7]	28 years	Male	Progressive lower limb weakness
Alla [11] (article in French)	64 years	Male	Radiculitis features followed by cauda equina syndrome features
Lee [8]	4 years	Female	Cauda equina syndrome features (lower limb weakness and urinary retention)
Present case report	3 years	Male	Cauda equina syndrome features

However, spinal intramedullary metastasis of medulloblastoma rarely occurs. To date, there have been a limited number of cases with spinal intramedullary metastasis reported in the literature [12-14]. Zumpano, in 1978, reported on a patient with intramedullary metastatic medulloblastoma several weeks after resecting the posterior fossa tumor [12]. Subsequently, a similar case was described by Stanley *et al.* in 1986 [5]. Recently, Inoue and colleagues reported on a child who had progressive weakness as the initial presenting features for intramedullary spinal metastasis [14]. In general, medulloblastoma spinal metastasis varied from nodular lesions to complete spinal block [5]. Our patient had extramedullary nodules, leptomeningeal enhancement and spinal block at the lumbar region. The cranial MRI findings were of sufficient importance to alter the management of our patient and to pave the way for different treatment strategies.

## Conclusions

Cauda equina syndrome as the presenting clinical feature for medulloblastoma is rare. Similar clinical presentations in the pediatric age group should raise the suspicion of cranial neoplastic pathology. Cranial neuroimaging is important to rule out drop metastasis and to optimize patient management.

## Consent

Written informed consent was obtained from the patient’s legal guardian for publication of this case report and any accompanying images. A copy of the written consent is available for review by the Editor-in-Chief of this journal.

## Competing interests

The authors declare that they have no competing interests.

## Authors’ contributions

FA and AU analyzed and interpreted the patient clinical and radiological data and prepared the manuscript. HA performed the histopathological examination, prepared the pathology slides, and reviewed the manuscript. AA reviewed the manuscript. EA reviewed the manuscript and gave final input prior to publication. FA wrote the manuscript and prepared the radiology figures. All authors read and approved the final manuscript.
